# Temporal Trends in the Management of Midshaft Clavicle Fractures: A Systematic Review

**DOI:** 10.7759/cureus.96532

**Published:** 2025-11-10

**Authors:** Mohammed Sayed, Swastik Sutar, Fady Kamel, Aadil Faleel, Amitabh J Dwyer

**Affiliations:** 1 Trauma and Orthopaedics, Peterborough City Hospital, Peterborough, GBR; 2 Trauma and Orthopaedics, West Suffolk Hospital, Cambridge, GBR; 3 Trauma and Orthopaedics, North West Anglia NHS Foundation Trust, Huntingdon, GBR

**Keywords:** a systematic review, clavicle fracture fixation, conservative vs surgical management, functional outcome, mid-shaft clavicle fracture, non-operative management, operative fixation, temporal trend analysis, union rate

## Abstract

Midshaft clavicle fractures are among the most common shoulder girdle injuries and have traditionally been managed conservatively. Over the past two decades, evidence comparing operative and non-operative approaches has expanded, and this systematic review aimed to evaluate functional outcomes, union rates, complications, and temporal trends in management between 2005 and 2025. A comprehensive literature search was performed across PubMed, Embase, CINAHL, Web of Science, the Cochrane Library, and Ovid MEDLINE for studies published during this period. Randomised controlled trials (RCTs), cohort studies, comparative observational studies, and systematic reviews or meta-analyses directly comparing operative and non-operative management in adults were included. Two reviewers independently screened titles, abstracts, and full texts in accordance with Preferred Reporting Items for Systematic Reviews and Meta-Analyses (PRISMA) guidelines, and study quality was assessed using the Cochrane Risk of Bias 2 (RoB-2) tool for RCTs and the Risk of Bias in Non-randomised Studies of Interventions (ROBINS-I) tool for non-randomised studies. Grey literature and non-peer-reviewed reports were excluded to ensure methodological rigour.

A total of 28 studies met the inclusion criteria (15 RCTs and 13 cohort or observational studies), enrolling 3,094 patients. Additional systematic reviews and meta-analyses were examined to contextualise and support primary findings. The mean age ranged from 20 to 45 years, with a 70-80% male predominance. Road traffic accidents and sports injuries were the most frequent mechanisms, and follow-up duration ranged from 6 to 53 months. Operative management (n = 1,572) primarily involved plate fixation, whereas non-operative care (n = 1,522) utilised slings or figure-of-eight harnesses. Surgical fixation was consistently associated with shorter union times (16-18 vs. 24-30 weeks) and lower rates of non-union (0.8-2.4% vs. 11-23%) and malunion (0-4.5%, occurring only in conservative groups). Early and intermediate functional outcomes generally favoured surgery, but long-term results often converged with conservative treatment. Conservative management avoided implant-related complications but showed higher risks of non-union and malunion, whereas surgical complications were more frequent (16-40%), most commonly hardware irritation or infection. A temporal trend was observed - earlier studies (2007-2015) strongly supported surgical fixation, while more recent evidence (2020-2025) emphasises selective indications and shared decision-making.

Overall, evidence from 2005 to 2025 demonstrates a clear temporal evolution in the management of midshaft clavicle fractures. Surgical fixation provides faster recovery, earlier union, and lower non-union and malunion rates, particularly in young, active patients with displaced fractures. However, long-term functional outcomes frequently align with conservative care, which remains appropriate for low-demand patients. Current findings highlight the importance of an individualised, patient-centred approach informed by fracture pattern, activity level, and patient preference.

## Introduction and background

Clavicle fractures are among the most common shoulder girdle injuries, with the majority occurring at the midshaft [1-5]. Historically, these injuries were managed conservatively with slings or figure-of-eight bandages, based on the assumption of high union rates and satisfactory long-term outcomes. Over the past two decades, however, this approach has been increasingly questioned. Randomised controlled trials (RCTs) and large cohort studies have reported higher non-union and malunion rates with non-operative management, whereas surgical fixation, typically using locking plate fixation or intramedullary devices, has demonstrated faster union, improved early functional outcomes, and reduced deformity. Nonetheless, surgery is associated with implant-related complications, reoperation, and inconsistent long-term benefits, which limit its universal adoption [6,7]. Systematic reviews and meta-analyses have synthesised these findings, though conclusions vary by population and study period. Earlier evidence (2007-2015) generally favoured surgery, while more recent data (2020-2025) highlight the convergence of long-term outcomes and emphasise the importance of shared, individualised decision-making. Accordingly, this systematic review aims to evaluate current evidence comparing operative and non-operative management of midshaft clavicle fractures, focusing on functional outcomes, union rates, complications, and temporal trends over the past two decades.

Epidemiology

The clavicle is an S-shaped bone, typically longer, thicker, and more curved in males than in females, with the left clavicle usually longer than the right [8]. Its subcutaneous position makes it highly susceptible to trauma and among the most common skeletal injuries after distal radius fractures. Clavicle fractures represent 2.6-10% of all fractures and up to 44% of shoulder-related injuries [9-13]. They predominantly affect young and middle-aged adults, with an estimated 70% male predominance and a male-to-female ratio of approximately 2:1 [11]. Incidence peaks in the second and third decades of life in men and follows a bimodal distribution in women, with higher rates among the young and the elderly [4,13]. The annual incidence among adolescents and adults is estimated at 29-64 per 100,000 per year [4], with the incidence of open clavicle fracture being only 0.1% to 1% [11]. Due to their frequency and functional impact, particularly in working-age populations, clavicle fractures hold significant clinical and socioeconomic importance.

Fracture distribution and mechanisms of injury

Midshaft fractures comprise 69-82% of all clavicle fractures, with lateral and medial fractures representing 12-26% and 2-6%, respectively [3]. This distribution can be attributed to their anatomical differences. The medial and lateral parts of the clavicle are firmly secured by strong ligaments and muscles, whereas the midshaft lacks such reinforcement. Consequently, it is more prone to injury from direct or indirect trauma. Additionally, fracture displacement occurs due to muscular pull-superior and posterior rotation of the medial fragment by the sternocleidomastoid, and inferior displacement of the lateral fragment due to pectoral muscles and body weight. Common mechanisms include road traffic accidents, falls onto the shoulder, and sports-related injuries. These fractures often affect young active males, typically involving the dominant side. Indirect trauma, such as a fall onto an outstretched hand, is also common, reflecting the clavicle’s exposure to diverse loading forces in both everyday and high-impact activities.

Classification

Several classification systems exist, including those proposed by Neer and the AO Foundation, but Robinson’s system is the most widely used. It categorises fractures according to anatomical location, displacement, comminution, and articular involvement. This classification divides clavicle fractures into three main types: Type 1 (medial) fractures are subdivided into 1A, which are nondisplaced and extra-articular, and 1B, which are displaced or intra-articular. Type 2 (midshaft) fractures include 2A, which are nondisplaced or minimally displaced and 2B, which are displaced with wedge or comminuted fractures. Type 3 (lateral) fractures are classified as 3A, which are nondisplaced, and 3B, which are displaced with coracoclavicular ligament injury. Among midshaft fractures, displaced types (2B1 and 2B2) are the most clinically relevant due to their higher risk of non-union and functional impairment, particularly in active adults.

Management overview

Optimal treatment of displaced midshaft fractures remains debated. Historically, non-operative care was preferred because most fractures united even with displacement, and many non-unions were asymptomatic. Standard conservative methods include slings, collar-and-cuff supports, and figure-of-eight bandages [11]. However, non-operative management can lead to non-union or malunion rates as high as 15% and patient dissatisfaction of up to 31% [3]. Symptomatic non-union causes pain, weakness, and delayed return to work [11], while mean clavicular shortening of approximately 1.2 cm has been linked to reduced shoulder strength and endurance [7].

Surgical fixation using open reduction and internal fixation (ORIF) with pre-contoured S-shaped locking compression plates (LCPs) or intramedullary devices (e.g., Rush pins, Kirschner Wire (K-wires), or nails) has become increasingly popular for displaced and comminuted fractures. Absolute indications for surgical management include open or severely displaced fractures, comminution, skin tenting, clavicular shortening greater than 20 mm, angulation exceeding 30°, floating shoulder, or neurovascular compromise [12]. Relative indications include polytrauma, symptomatic malunion or non-union, and patients with high physical demands [12].

The 2007 Canadian Orthopaedic Trauma Society trial demonstrated that surgical fixation significantly reduced non-union and malunion rates and improved functional outcomes at one year [3]. In athletes, conservative management is linked to delayed recovery and poorer return-to-sport performance due to shortening and malunion. While surgery offers faster recovery and anatomical alignment, its risks, such as infection, neurovascular injury, and hardware irritation, should be carefully weighed when selecting patients. To address these ongoing uncertainties, this review systematically examines the available literature to identify the best evidence for managing midshaft clavicle fractures and to evaluate how treatment practices have evolved over the past two decades.

Aims of the study

This review aims to comprehensively evaluate the efficacy, complications, and functional outcomes associated with both operative and non-operative management of midshaft clavicle fractures. It further seeks to determine whether recent evidence supports surgical or conservative treatment as the preferred approach, and to assess how management practices and clinical outcomes have evolved over the past two decades.

## Review

Methods

Search Strategy

**Figure 1:**
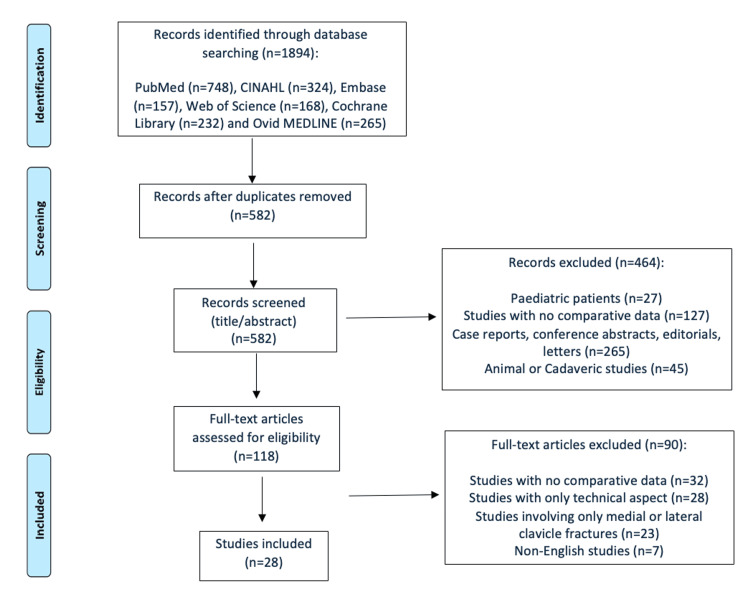
PRISMA Flow Diagram PRISMA: Preferred Reporting Items for Systematic Reviews and Meta-Analyses

A systematic literature search was conducted across PubMed, CINAHL, Embase, Web of Science, the Cochrane Library, and Ovid MEDLINE for studies published between January 2005 and June 2025. The review followed the Preferred Reporting Items for Systematic Reviews and Meta-Analyses (PRISMA) 2020 guidelines (Figure 1). Keywords included terms related to midshaft clavicle fractures; operative management, such as ORIF, plate fixation, intramedullary nails, Rush pins, and Kirschner wires; and non-operative management, including conservative treatment, sling immobilisation, collar-and-cuff, and figure-of-eight bandages. Additional search terms captured relevant outcome measures, including union rates, complications, and functional outcomes, such as the Constant-Murley Score (CMS) and Disabilities of the Arm, Shoulder, and Hand (DASH) score. Eligible study designs included RCTs, prospective and retrospective cohort studies, meta-analyses, and systematic reviews. Reference lists of included studies and relevant reviews were manually screened to ensure comprehensive coverage. Grey literature, including conference abstracts and unpublished manuscripts, was excluded to maintain methodological quality and reproducibility.

Study Selection

All search results were imported into a reference manager, and duplicates were removed. Two reviewers (SS, MS) independently screened titles, abstracts, and full texts in three stages. Discrepancies at the title or abstract stage were carried forward to full-text review. Any disagreements during full-text assessment were resolved by consensus or through consultation with a senior reviewer (AF).

Studies were eligible for inclusion if they examined adult patients (≥16 years) with midshaft clavicle fractures and provided comparative data between operative and non-operative management. Eligible designs included RCTs, prospective or retrospective cohort studies, and systematic reviews or meta-analyses. Studies were required to report clinically relevant outcomes, including pain, fracture union, complications, or functional recovery. Only English-language articles published between 2005 and 2025 were considered in this study.

Studies were excluded if they involved paediatric patients (<16 years), investigated only medial or lateral third clavicle fractures, or focused solely on the technical aspects of surgery without reporting clinical outcomes. Additional exclusion criteria included pathological or open fractures, animal or cadaveric studies, and non-comparative publications such as case reports, conference abstracts, editorials, or letters. The full screening process is illustrated in the PRISMA flow diagram (Figure 1). High-quality systematic reviews and meta-analyses were considered separately to contextualise and support the findings of primary studies.

Data Extraction and Analysis

Two reviewers (SS, MS) independently extracted data using a predefined Microsoft Excel sheet (Microsoft Corporation, Redmond, WA, USA). Extracted variables included study design, population demographics, intervention and comparator arms, follow-up duration, functional scores (e.g., CMS, DASH), union rates, and complications. No identifying or patient-level data were collected. Data accuracy was verified through cross-checking and random spot-checking. Findings were summarised descriptively, as this review focused on a narrative synthesis rather than quantitative pooling. The study characteristics are summarised in Table 1 for RCTs and cohort studies and Table 2 for systematic reviews and meta-analyses. Reported statistical tests and p-values were extracted directly from the original studies, which used their respective analytical methods; no new statistical analyses were performed in this review.

**Table 1:** Characteristics of Included RCTs and Cohort Studies Reported p-values are presented exactly as stated in the original studies, with p < 0.05 considered statistically significant unless otherwise specified. Functional outcome measures include the Constant-Murley Score (CMS), Disabilities of the Arm, Shoulder, and Hand (DASH), and the University of California, Los Angeles Shoulder Score (UCLA). All values represent results extracted directly from published data; no additional statistical analysis was performed. CSS: Constant Shoulder Score; SSV: subjective shoulder value; CRPS: complex regional pain syndrome; RCT: randomised controlled trial; LCP: locking compression plate; ORIF: open reduction and internal fixation; TENS: Titanium Elastic Nail System; N: sample size (number of patients); n: number of participants in each treatment arm; P: P-value (statistical significance)

Study	Year	Design	N	Intervention	Comparator	Follow-Up (Months)	Main Functional Outcomes	Union/Complications
Melean et al. [1]	2015	RCT	76	LCP/recon plates (n = 34)	Sling (n = 42)	12	Higher Constant Shoulder Score (CSS) at 6 months (85 vs 81; p = 0.004) & 12 months (93 vs 87; p = 0.003) in surgical group.	Higher union rates in surgical group at 6 weeks (24.1% vs 5.3%; p = 0.005) and 12 weeks (81% vs 16.7%; p = 0.004). 4 non-unions in non-operative group vs 0 in operative group.
Ma et al. [2]	2020	Cohort	85	External Fixation (n = 25)/Plate (n = 30)	Sling (n = 30)	32	Better CSS/DASH in operative group	Faster union in operative group (10–12 vs 16 weeks); Non-union (1 vs 3) and malunion (1-3 vs 12) higher in non-operative group.
Canadian Orthopaedic Trauma Society (COTS) [3]	2007	RCT	132	Superior LCP/recon plates (n = 67)	Sling (n = 65)	12	Better DASH/CSS in surgical group (p < 0.01)	Faster union (16 vs 28 weeks) in surgical group. 2 non-unions in the operative group vs 7 in non-operative group.
Jeswani et al. [4]	2025	Cross-sectional	60	K-wire (n = 30)	Sling (n = 30)	6	Better DASH in surgical group (p<0.05)	Union: 93.3% vs 73.3% (p<0.05). Non-union: 3.3% vs 16.7% (p<0.05). Malunion: 3.3% vs 10% (p<0.05)
Han et al. [5]	2024	Cohort	105	LCP (n = 50)	Figure-of-eight brace (n = 55)	20	No significant difference in DASH/CMS operative group showed earlier return to work, range of motion, and strength recovery	Union rate 98% vs 87.3%; mean union time 2.37 vs 3.69 months; malunion occurred only in the conservative group; operative group had transient numbness (~46%) and frequent implant removal (~90%).
Gautam et al. [6]	2023	Observational	48	LCP (n = 20)	Figure-of-eight brace (n=28)	12	Better University of California, Los Angeles Shoulder Score (UCLA)/ DASH in conservative group (p<0.05)	95% union rate in surgical vs 89% in non-operative group. Few non-union in operative group (4.55% vs 10.71%).
Ashraf et al. [7]	2023	Comparative	120	Plate (n = 60)	Brace/pouch (n = 60)	3	Better CSS in operative group (p<0.05)	Higher complication rates, including muscle wasting and complex regional pain syndrome (CRPS), in the surgical group.
Gholap et al. [8]	2023	Cross-sectional	52	LCP (n = 26)	Figure-of-eight bandage/collar and cuff (n = 26)	6	No significant difference in CMS at 6 months (p > 0.05), but earlier recovery and better early satisfaction in surgical group	Faster union (2.98 vs 5 months, p < 0.05); 0 non-union and 0 malunion in surgical vs 1 non-union and 3 malunion in conservative group; overall complications comparable (p > 0.05).
Kumar and Jee [9]	2023	Cross-sectional	100	LCP (n = 50)	Sling/brace (n = 50)	Not reported	Higher satisfaction in surgical group	Fewer complications in operative group (4 vs 20). Faster union (2.9 vs 5 months).
Mankar et al. [10]	2023	RCT	30	Intramedullary screw nail (n = 15)	Figure-of-eight brace (n = 15)	6	University of California, Los Angeles Shoulder Score (UCLA) significantly higher in operative group at 1 month (24.27 vs. 16.4; p = 0.0001), 3 months (27.64 vs 25.47; p = 0.0524) & 6 months (31.54 vs. 29.27; p = 0.0003)	Faster union in surgical group (1.73 vs 2.5 months). 1 non-union in both groups.
Kumar and Prakash [11]	2023	Cohort	140	LCP (n = 70)	Sling/brace (n = 70)	Not reported	Better functional recovery in surgical group	Faster union (3.8 vs 6 months). Complication rate similar in both groups.
Murray et al. [12]	2022	Cohort	347	Early/late fixation (n = 52)	Sling (n = 306)	12	Not reported	6 non-union (2.7%) and 1 malunion (0.5%) in non-operative vs 0 in operative group
Kumar et al. [13]	2022	RCT	50	TENS (n = 25)	Sling (n = 25)	Not reported	Higher CSS in operative group (23 vs 15)	Faster union in operative group (1.7 vs 2.5 months)
Ahrens et al. [14]	2017	RCT	301	ORIF precontoured plate (n = 154)	Sling (n = 147)	9	DASH/CSS better at 6 weeks (p < 0.001) and 3 months (p = 0.023) in operative group; no difference at 9 months.	No difference in non-union or malunion (28% vs 27%) at 3 months. Non-union less in operative group (0.8% vs 11%) at 9 months.
Virtanen et al. [15]	2012	RCT	60	Plate (n = 28)	Sling (n = 32)	12	No difference in DASH/Constant scores (p > 0.05)	Fewer non-union in operative group (0% vs 24%)
Tamaoki et al. [16]	2017	RCT	117	Plate (n = 59)	Figure-of-eight harness (n = 58)	12	No difference in DASH scores (p > 0.05)	Lower non-union rates in surgical group (0% vs 14.9%). More paraesthesia in surgical group (13.7% vs. 2.1%)
Kumar et al. [17]	2022	Cohort	40	LCP (n = 15)	Figure-of-eight brace brace (n = 25)	12	No significant difference in DASH/UCLA scores	Operative group had faster union (60% vs 28% in 12 weeks). Higher complication in operative group (9 vs 2).
Reddy et al. [18]	2025	Cohort	111	Dual mini-fragment plates (n = 62)	Sling (n = 49)	40.8	No Subjective Shoulder Value (SSV)/pain difference (p>0.5)	Faster union in operative group (20.8 weeks vs 28.6 weeks); fewer non-union in operative group (0% vs 8%)
Woltz et al. [19]	2017	RCT	160	Plate (n = 86)	Sling (n = 74)	12	No difference in DASH/Constant scores (p > 0.05)	Non-union 2.4% vs 23.1%
Biz et al. [20]	2023	Cohort	134	LCP (n = 59)	Figure-of-eight bandage (n = 75)	29.6	Better CSS in conservative group (96.77 vs 93.53). Surgical group had longer return to sports (4.93 vs 4.08 months).	0 non-union in both groups. 8 re-fractures in non-operative group. 5 infections in surgical group.
Woltz et al. [21]	2018	Follow-up RCT	79	Plate (n = 40)	Sling (n = 39)	53	No long-term functional difference	Not reported
Andrade-Silva et al. [22]	2015	RCT	59	Plate (n = 33)	Elastic nail (n = 26)	12	No difference in DASH/Constant scores (p > 0.05)	More implant-related pain in nail group. Time to union was similar (16.8 vs 15.9 weeks, p = 0.352).
Robinson et al. [23]	2013	RCT	200	Superior LCP (n = 95)	Collar and cuff (n = 105)	12	Better DASH (3.4 vs 6.1) /CSS (92 vs 87.8) in surgical group	Fewer non-union in operative group (1 vs 16)
Bhardwaj et al. [24]	2018	RCT	69	Superior LCP (n = 36)	Sling (n = 33)	24	Better DASH/CSS (89.42 vs 76.24) in surgical group	Time to union 15 vs 23 weeks; fewer non-union in surgical group
Shetty et al. [25]	2017	Cohort	30	LCP (n = 16)	Brace (n = 14)	6	No significant difference in DASH scores (8.57 vs 7.75)	6 malunions in non-op vs 0 in operative group. 0 non-union in both groups.
Ban et al. [26]	2021	RCT	120	Plate (n = 60)	Sling (n = 60)	12	Significant DASH in operative group at 6 weeks. (p = 0.001). No significant difference (p = 0.277 for DASH and p = 0.184 for CSS) at 12 months	Significantly higher rate of non-union in non-operative group (p = 0.014)
Altamimi et al. [27]	2008	RCT	132	Superior plate (n = 67)	Sling (n = 65)	12	Better DASH/CSS in surgical group (p = 0.001)	Faster union rate in operative group (16.4 vs 28.4); Lower non-union rate in operative group (1.2% vs 17%)
Cole et al. [28]	2014	RCT	200	Superior LCP (n = 95)	Sling (n = 105)	12	Significantly better CSS (92.0 vs 87.8; p = 0.01) and DASH (3.4 vs 6.1; p = 0.04) in operative group at 12 months.	Lower non-union in operative group (1% vs 17%)

**Table 2:** Included Systematic Reviews and Meta-Analyses RCT: randomised controlled trial; DASH: Disabilities of the Arm, Shoulder and Hand

Author	Year	Type	Studies Included	Main Findings
Yan et al. [29]	2022	Meta-analysis	31 RCTs	Surgery reduces non-union rate and bone-related complications, showing DASH and Constant benefits
Qvist and Jensen [30]	2024	Meta-analysis	10 RCTs	Early DASH benefit with surgery, small differences in surgery vs. conservative at 3-6 months

Quality Assessment

Methodological quality was assessed independently by two reviewers. RCTs were evaluated using the Cochrane Risk of Bias 2 (RoB-2) tool, and non-randomised studies were assessed using the Risk of Bias in Non-randomised Studies of Interventions (ROBINS-I) tool. Discrepancies were resolved by discussion and consensus. Formal quantitative scoring was not performed, as this review provided a narrative synthesis rather than a meta-analysis; however, the appraisal ensured that included studies were of moderate to high methodological quality. Grey literature and non-peer-reviewed studies were excluded to preserve rigour and reproducibility.

Results

Study Characteristics

A total of 28 primary studies were included in this systematic review: 15 RCTs and 13 cohort or observational studies, enrolling a total of 3,094 patients. The mean patient age across studies ranged from 20 to 45 years, with a consistent male predominance (70-80%).

The most common mechanism of injury was road traffic accidents (RTAs), accounting for the majority of cases in large cohort studies such as Kumar et al. [13] and Jeswani et al. [4]. Sports-related injuries, particularly cycling and contact sports, were the next most common, followed by falls, which were more frequently reported in older age groups. Several studies noted that injuries often involved the dominant side and were more prevalent among young, active males.

Follow-up durations ranged from 6 to 53 months, enabling assessment of both short- and long-term outcomes. Of the total patient cohort, 1,572 underwent operative management-most with plate fixation (n = 1,160) via superior, anterosuperior, or anterior approaches. Intramedullary fixation was used in 412 patients, with devices including the Titanium Elastic Nail (TEN™) System, Rockwood pins, and threaded intramedullary screws. Non-operative management (n = 1,522) commonly utilised sling immobilisation (n = 1,035) or figure-of-eight harnesses (n = 487).

For clarity, the studies are presented in two separate tables: Table 1 summarises the characteristics and outcomes of the primary studies (RCTs and cohort/observational designs), and Table 2 presents the included systematic reviews and meta-analyses, which provide pooled evidence to contextualise the findings of individual trials. This division allows a clearer distinction between direct trial data and higher-level synthesised evidence.

Functional Outcomes

The DASH score evaluates upper limb function, where lower scores indicate better performance [3]. Surgical management was associated with lower DASH scores, reflecting better functional recovery compared with conservative treatment. This improvement was statistically significant in multiple RCTs, particularly at 6-12 months. In the early period (three months), differences were small and inconsistent, with some studies showing modest benefits for surgery, while others reported no significant difference. During the intermediate phase (6-12 months), surgery provided a measurable advantage, with improved DASH scores in several RCTs, such as Ahrens et al. [14] and Altamimi and McKee [27]. At late follow-up (>24 months), the difference became more pronounced, as operative patients demonstrated significantly better function and sustained benefits at long-term assessment.

**Figure 2:**
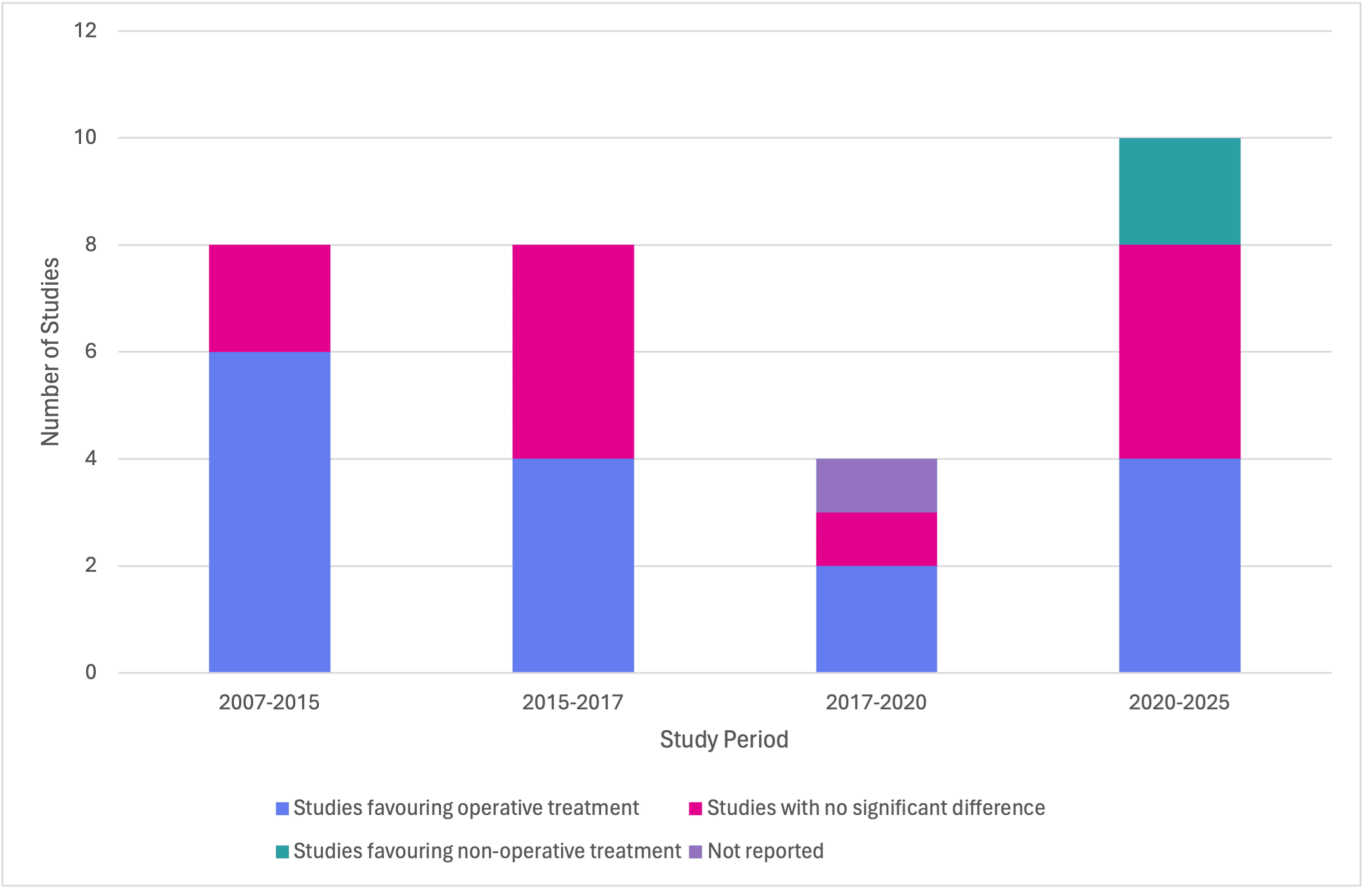
Temporal Trends in Functional Outcomes Early studies (2007-2015) predominantly favoured surgery (six supported operative management; two found no difference). Mid-period results (2015-2020) were mixed, while recent studies (2020-2025) show heterogeneity, with some favouring conservative care (four favoured surgery, four found no difference, two supported non-operative care).

The CMS is a 100-point scale that assesses pain and daily functional capacity, where higher scores reflect superior recovery [3]. Operative patients demonstrated higher Constant scores, indicating superior shoulder strength, mobility, and pain relief. At early and intermediate follow-up, the results were variable across studies, with some reporting no difference between groups. However, by 24 months, surgical cohorts consistently achieved clinically meaningful improvements, and several trials demonstrated significant advantages over conservative care. Overall, these functional outcome measures suggest that although both approaches may achieve satisfactory recovery by one year, surgery accelerates recovery and offers superior long-term function, particularly in young and active patients. These overall patterns and the temporal evolution of functional outcomes between 2007 and 2025 are illustrated in Figure 2.

Time to Union

**Figure 3:**
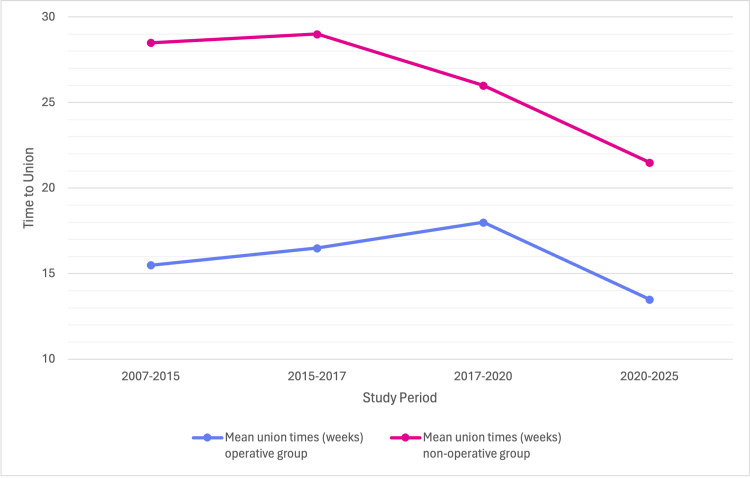
Temporal Trends in Time to Union Across all periods, surgery achieved faster union (≈13-18 weeks) compared with non-operative treatment (≈21-30 weeks), though the difference has narrowed in recent studies (2020-2025). Overall, the evidence indicates that operative fixation shortens time to union by approximately 6-10 weeks relative to non-operative care.

Across the included studies, surgical intervention consistently shortened the time to radiographic union compared with non-operative management. Early RCTs (e.g., Altamimi et al. [27], Ahrens et al. [14]) reported mean union times of 16-18 weeks in surgical groups versus 24-30 weeks in non-operative groups, a difference associated with faster early recovery. Later multicentre studies, such as Woltz et al. [19,21] and Robinson et al. [23], confirmed this finding, noting earlier union and fewer non-unions with plating, although functional outcomes at one year were similar between groups. More recent investigations (e.g., Mankar et al. [10], Kumar et al. [13]) highlighted the role of intramedullary devices, which achieved union in as little as 10-12 weeks compared with 20-28 weeks for conservative care. These techniques, however, were associated with implant-related complications such as irritation and hardware migration. The comparative progression of mean union times across studies and treatment methods over the past two decades is shown in Figure 3. Overall, the evidence indicates that operative fixation shortens time to union by approximately 6-10 weeks relative to non-operative care. This advantage frequently translates into an earlier return to work and physical activity, as reported in multiple RCTs and cohort studies (Tables 1, 2).

Complications

**Figure 4:**
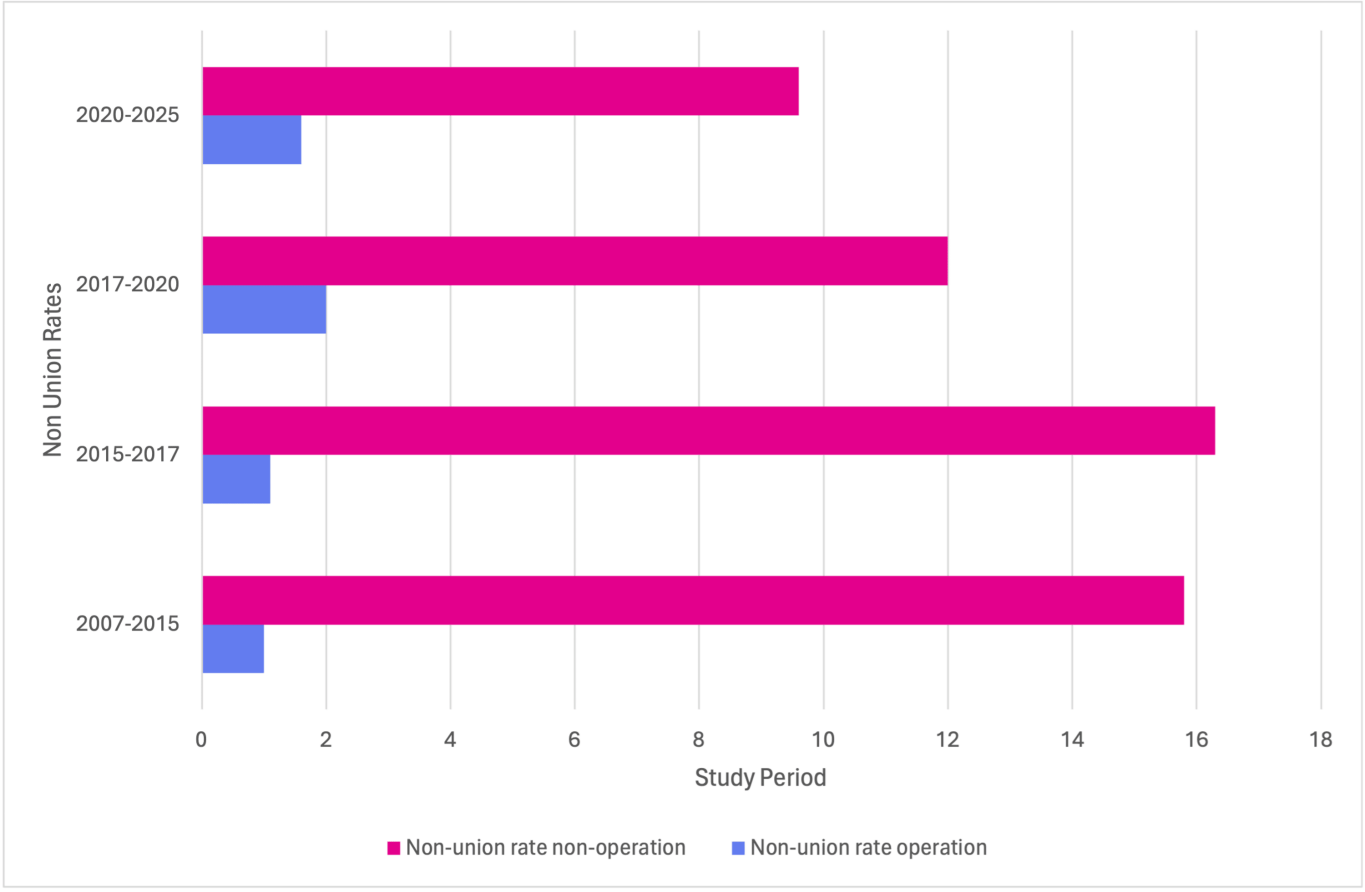
Temporal Trends in Non-union Rates Operative management consistently maintained very low rates (0.8-2%), while non-operative treatment declined from 15% to 16% in early trials to ~10% in recent years.

The complication profile differed markedly between treatment strategies. Surgical treatment markedly reduced non-union rates (0.8-2.4%) compared with non-operative management (11-23%). Several high-quality RCTs consistently reported lower non-union rates in operative arms. The evolution of non-union rates across studies and treatment eras is summarised in Figure 4. Malunion was rare with surgical fixation (0-4.5%) but frequent in conservatively treated groups, with some studies reporting malunion in all conservatively managed displaced fractures, particularly when shortening exceeded 1.5 cm. Implant-related complications such as hardware irritation, superficial infection, and implant failure​​​ were more common in surgical groups (16-40%). Most were resolved with elective hardware removal and did not compromise final outcomes. Conservative care was associated with a higher incidence of cosmetic dissatisfaction, shoulder asymmetry, and residual deformity, while surgical groups experienced higher rates of paraesthesia and implant-related symptoms.

Discussions

Early Evidence (2007-2015)

High-quality RCTs during this period strongly favoured operative fixation, demonstrating superior function, faster union, and lower non-union rates. Multiple RCTs [1,3,23,28] reported significantly better functional outcomes (DASH and Constant scores), earlier fracture union, and fewer non-unions following surgery compared with conservative care. Specifically, the Canadian Orthopaedic Trauma Society trial [3] showed shorter time to union (16 vs. 28 weeks) and lower non-union (1-1.2% vs. 15-17%) after plating. Robinson et al. [23] similarly found non-union (1% vs. 16%) in favour of surgery, with better early DASH or Constant scores and earlier union (16-18 vs. 24-30 weeks). Melean et al. [1] reported higher Constant scores at six months (p = 0.004) and 12 months (p = 0.003), greater CT-confirmed union at 12 weeks (81% vs. 16.7%), faster return to work (2.9 vs. 3.7 months; p = 0.003), and fewer non-unions (0 vs. 4) with surgery. Altamimi et al. [27] further noted greater cosmetic satisfaction among surgically treated patients. Collectively, these findings established surgery as the preferred approach for displaced midshaft fractures, particularly in young and active individuals.

Transitional Period (2015-2017)

Subsequent studies produced more variable results. Surgery continued to demonstrate lower rates of non-union and malunion but carried increased risks of implant-related complications. Final functional outcomes often converged with those of non-operative treatment. Andrade-Silva et al. [22] found no significant difference in functional outcomes (DASH or Constant scores) at six or 12 months (p > 0.05), with comparable union times (16.8 vs. 15.9 weeks; p = 0.352). However, surgical patients experienced higher implant-related pain (40% vs. 14%; p = 0.035) and greater reoperation rates (72% vs. 3%; p < 0.001). Similarly, Ahrens et al. [14] observed improved early DASH and Constant scores (six weeks and three months; p < 0.05) but no difference by nine months, although the surgical group maintained a lower non-union rate (0.8% vs. 11%; p < 0.001). Virtanen et al. [15] and Tamaoki et al. [16] reported similar findings, with no functional differences at one year but fewer non-unions with surgery. Shetty et al. [25] also observed no significant difference in DASH scores at 24 weeks (p = 0.861), though malunion was higher in the conservative group (6 vs. 0 cases).

Refinement of Indications (2017-2020)

Later evidence began stratifying outcomes by fracture pattern and patient profile. Surgery appeared most beneficial for displaced fractures and high-demand patients rather than all midshaft fractures. Bhardwaj et al. [24] reported superior functional scores (DASH and Constant Shoulder Score (CSS)) and faster union with surgery (15.6 vs. 22.8 weeks). Woltz et al. [19,21] found no long-term functional differences at one year but greater patient satisfaction with surgery (88% vs. 41%). Non-union rates remained significantly lower with plating (2.4% vs. 23.1%; p < 0.0001). Ma et al. [2] compared plating and external fixation with conservative care, reporting quicker healing, higher satisfaction, and improved union rates with surgical methods, albeit with more implant-related irritation.

Recent Evidence (2020-2025)

Recent studies demonstrate that non-operative care can achieve comparable long-term function in appropriately selected patients, though non-union remains more common. Surgery continues to deliver faster recovery and more reliable union, but at the expense of hardware complications. Biz et al. [20] reported higher Constant scores with conservative care (96.77 vs. 93.53; p < 0.0001) but increased re-fracture rates (8 vs. 0). In contrast, Mankar et al. [10] found quicker union, improved University of California, Los Angeles Shoulder Score (UCLA) scores at one and six months, and fewer complications with surgery. Jeswani et al. [4] observed reduced non-union with intramedullary fixation (3.3% vs. 16.7%; p < 0.05) but higher revision rates. Han et al. [5] observed equivalent QuickDASH scores at two years but universal malunion in the conservative group (100%). Reddy et al. [18] also confirmed advantages in time to union (20.8 vs. 28.6 weeks; p < 0.001) and fewer non-unions (0% vs. 8.2%; p = 0.035) after surgery, though pain and subjective function were comparable at 3.4 years (p > 0.05).

Strengths and limitations

This review provides a broad synthesis of evidence spanning two decades, integrating both primary studies and high-level meta-analyses to assess evolving management trends. The inclusion of temporal stratification offers insight into shifting clinical practice and evidence-based decision-making.

Despite these strengths, heterogeneity among included studies - particularly in study design, sample size, follow-up duration, and outcome - limited direct comparison and prevented meta-analytical pooling. Most studies originated from high-income countries, potentially limiting generalisability to low- and middle-income populations. Reporting of complications was also inconsistent, underscoring the need for standardised outcome reporting in future research.

## Conclusions

Surgical management of midshaft clavicle fractures generally results in faster recovery, shorter time to union (16-18 weeks vs. 24-30 weeks), and lower risks of non-union and malunion. Functional outcomes, measured by DASH and CMS, typically favour surgery in the early and intermediate phases (up to 12 months) but often converge with non-operative care at longer-term follow-up (one to two years). Conversely, conservative treatment carries higher rates of non-union (11-23%) and malunion but avoids hardware-related complications such as irritation, infection, and the need for implant removal. Surgical management, in contrast, is associated with implant-related complications in 16-40% of cases, though most are manageable with secondary procedures.

A clear temporal trend is evident - earlier studies favoured routine surgical fixation, while recent evidence supports selective intervention and shared decision-making. Surgery remains most beneficial for young, active individuals with displaced fractures, while conservative management may provide excellent outcomes in low-demand patients. Future research should refine surgical indications, optimise patient selection, and assess outcomes across broader, more diverse populations.
